# The RAPID-CTCA trial (Rapid Assessment of Potential Ischaemic Heart Disease with CTCA) — a multicentre parallel-group randomised trial to compare early computerised tomography coronary angiography versus standard care in patients presenting with suspected or confirmed acute coronary syndrome: study protocol for a randomised controlled trial

**DOI:** 10.1186/s13063-016-1717-2

**Published:** 2016-12-07

**Authors:** Alasdair J. Gray, Carl Roobottom, Jason E. Smith, Steve Goodacre, Katherine Oatey, Rachel O’Brien, Robert F. Storey, Lumine Na, Steff C. Lewis, Praveen Thokala, David E. Newby

**Affiliations:** 1 Department of Emergency Medicine, Emergency Medicine Research Group, Royal Infirmary of Edinburgh, Edinburgh, UK; 2 University of Edinburgh, British Heart Foundation, Centre for Cardiovascular Science, Edinburgh, UK; 3 Edinburgh Clinical Trials Unit, Usher Institute, University of Edinburgh, Edinburgh, UK; 4 Plymouth University Peninsula Schools of Medicine and Dentistry, Plymouth, UK; 5 Derriford Hospital, Plymouth Hospitals NHS Trust, Plymouth, UK; 6 Academic Department of Military Emergency Medicine, Royal Centre for Defence Medicine (Research & Academia), Birmingham, UK; 7 School of Health and Related Research (ScHARR), University of Sheffield, Sheffield, UK; 8 Department of Infection, Immunity and Cardiovascular Disease, University of Sheffield, Sheffield, UK

**Keywords:** Acute coronary syndrome, Chest pain assessment, CT coronary angiogram, Cardiac CT

## Abstract

**Background:**

Emergency department attendances with chest pain requiring assessment for acute coronary syndrome (ACS) are a major global health issue. Standard assessment includes history, examination, electrocardiogram (ECG) and serial troponin testing. Computerised tomography coronary angiography (CTCA) enables additional anatomical assessment of patients for coronary artery disease (CAD) but has only been studied in very low-risk patients. This trial aims to investigate the effect of early CTCA upon interventions, event rates and health care costs in patients with suspected/confirmed ACS who are at intermediate risk.

**Methods/design:**

Participants will be recruited in about 35 tertiary and district general hospitals in the UK. Patients ≥18 years old with symptoms with suspected/confirmed ACS with at least one of the following will be included: (1) ECG abnormalities, e.g. ST-segment depression >0.5 mm; (2) history of ischaemic heart disease; (3) troponin elevation above the 99^th^ centile of the normal reference range or increase in high-sensitivity troponin meeting European Society of Cardiology criteria for ‘rule-in’ of myocardial infarction (MI). The early use of ≥64-slice CTCA as part of routine assessment will be compared to standard care. The primary endpoint will be 1-year all-cause death or recurrent type 1 or type 4b MI at 1 year, measured as the time to such event. A number of secondary clinical, process and safety endpoints will be collected and analysed. Cost effectiveness will be estimated in terms of the lifetime incremental cost per quality-adjusted life year gained. We plan to recruit 2424 (2500 with ~3% drop-out) evaluable patients (1212 per arm) to have 90% power to detect a 20% versus 15% difference in 1-year death or recurrent type 1 MI or type 4b MI, two-sided *p* < 0.05. Analysis will be on an intention-to-treat basis. The relationship between intervention and the primary outcome will be analysed using Cox proportional hazard regression adjusted for study site (used to stratify the randomisation), age, baseline Global Registry of Acute Coronary Events score, previous CAD and baseline troponin level. The results will be expressed as a hazard ratio with the corresponding 95% confidence intervals and *p* value.

**Discussion:**

The Rapid Assessment of Potential Ischaemic Heart Disease with CTCA (RAPID-CTCA) trial will recruit 2500 participants across about 35 hospital sites. It will be the first study to investigate the role of CTCA in the early assessment of patients with suspected or confirmed ACS who are at intermediate risk and including patients who have raised troponin measurements during initial assessment.

**Trial registration:**

ISRCTN19102565. Registered on 3 October 2014. ClinicalTrials.gov: NCT02284191.

## Background

Emergency department (ED) attendances with chest pain requiring assessment for acute coronary syndrome (ACS) are a major global health issue. In the USA there are eight million attendances annually with chest pain [1], and yet most are ultimately discharged home without a definitive diagnosis [2]. In the UK approximately 700,000 patients present annually to EDs with chest pain in England and Wales, resulting in around 350,000 emergency admissions [3]. Most of these patients present with, and are subsequently admitted for, evaluation of suspected ACS. This is becoming an increasing problem; chest pain admissions have doubled in the last decade, accounting for approximately 5% of all emergency admissions (the most common reason for acute hospital admission), whilst those for angina or ACS have fallen [4, 5]. Therefore, most patients admitted with suspected ACS are discharged without the condition being confirmed by subsequent investigation. Despite this, confirmed ACS remains a common diagnosis and is associated with major adverse outcomes.

### Diagnostic pathways for suspected ACS

Due to the consequences of inadvertent discharge of a patient with missed ACS and the limitations of initial clinical assessment, most patients with suspected ACS will require diagnostic investigation and a short hospital admission or a period of observation in the ED. This assessment and evaluation period in the UK is based on national and international guidelines [6–8] and includes serial cardiac biomarkers, typically troponin I or T, and a 12-lead electrocardiogram (ECG). Many troponin assays do not reach maximal sensitivity until 12 hours after chest pain onset, although newer high-sensitivity assays are increasingly used and recommended in contemporary guidelines [8]. Current European guidelines suggest presentation and 3-hour measurements of high-sensitivity troponin to rule in or rule out myocardial infarction (MI) in the ED, depending on the time since chest pain onset. This is supported by recent large cohort studies, assessing the diagnostic characteristics of high-sensitivity troponin at the limit of detection, showing exceptional negative predictive value for presentation samples [9]. These novel, highly sensitive troponin assays, along with suggested lower diagnostic and sex-specific thresholds of contemporary assays [10–12], will increase the number of patients with an elevated troponin result. Patients with an elevated troponin who present with suspected ACS will have sustained an acute MI according to the Universal Definition of MI [13]. Current recommendations are that these individuals should receive relevant management including invasive coronary angiography (ICA) within 24 hours of diagnosis [8], leading to a significant number of patients internationally requiring transfer to a percutaneous coronary intervention (PCI) centre for continued care. A further potential disadvantage of highly sensitive troponin assays and lower diagnostic thresholds of contemporary assays is the increased number of patients who have raised troponins but do not have coronary thrombosis and therefore do not require potent antithrombotic therapy and ICA. These may be related to cardiac (myocarditis, arrhythmia, stress-induced cardiomyopathy) or other conditions such as pulmonary embolism as well as type 2 MI [8, 12]. The unanticipated consequence of this may be increased numbers of patients receiving further investigation for ACS including ICA with consequent patient distress, risk and cost. Subsequent assessment to further delineate ischaemic heart disease, especially if ECG and troponin testing are negative, includes functional or anatomical testing. Further investigations to delineate future prognosis rather than immediate diagnosis are inconsistent in the UK [14], resulting in many patients being discharged from hospital with ’troponin-negative chest pain’ and no clear alternative diagnosis. This leads to many patients and clinicians feeling unclear about what to do if the patient has recurrent symptoms, since coronary artery disease (CAD) has not been unequivocally excluded. Elsewhere, ancillary testing during or soon after the ED attendance is widely adopted, leading to prolonged lengths of stay and significant cost [2].

ICA is recommended by the European Society of Cardiology (ESC) in confirmed ACS or for those patients believed to be at high risk of obstructive CAD, but it is costly and associated with a small but significant major complication rate, including death [8, 14]. It often requires the transfer of patients between hospitals; e.g. in the UK only around 35% of acute hospitals have on-site revascularisation facilities [15]. It is unknown how many patients receive unnecessary ICA, but it is likely to be a significant and potentially increasing number, if all patients with a raised troponin and chest pain receive ICA. Some patients with confirmed ACS do not receive ICA due to limited availability, belief that troponin elevation is due to an alternative condition or other reasons for a decision to pursue non-invasive management. It could be argued that the benefits of an invasive approach have not been established in patients who would have been deemed low risk before the advent of high-sensitivity troponin assays. On this basis, patients with suspected ACS could be investigated by CTCA, with onward referral for PCI or coronary artery bypass graft (CABG) surgery limited to patients with clearly treatable coronary obstruction. Indeed, CTCA has a similar discriminatory value in determining the need for coronary revascularisation as ICA [16].

### CT coronary angiography in chest pain assessment

CTCA has the potential to be a quicker, simpler, substantially cheaper and more readily delivered alternative to ICA and should translate into a highly effective and safe imaging strategy. A systematic review of 21 diagnostic accuracy studies of CTCA reported a pooled sensitivity of 99% and a specificity of 89% for detection of CAD [17]. A further recent meta-analysis of eight diagnostic cohort studies of CTCA in suspected ACS [18] reported a sensitivity of 94% (95% predictive interval 61–99%) and a specificity of 87% (95% predictive interval 16–100%), but decision analysis modelling was unable to draw reliable conclusions about the clinical and cost effectiveness of CTCA in suspected ACS. Three recent trials from the USA investigating CTCA in patients with chest pain presenting to the ED promote its use and widespread adoption [19–21]. Meta-analyses of four trials [22, 23] conclude that CTCA is safe, cost effective and associated with reduced hospital length of stay in the US health care system. However, the clinical event rates in these studies are low, with no difference between trial arms. Moreover, the participants had relatively long hospital stays and many additional tests compared with other international health care settings.

CTCA enables non-invasive anatomical quantification of CAD [2]. This allows accurate identification of patients who may benefit from coronary revascularisation [16] and more accurately targets patients for primary or secondary therapies, thus improving clinical outcomes. In those patients without disease, it may reduce hospital stay and recurrent hospitalisation as well as improve patient satisfaction due to clarity on the absence of CAD. However, if CTCA use results in an increase in ICA as a result of false positive or equivocal results in low-risk patients, it may increase the cost and risk without clinical benefit.

Since the start of the Rapid Assessment of Potential Ischaemic Heart Disease with CTCA (RAPID-CTCA) trial, five trials investigating the role of CTCA in stable CAD (the PROMISE, SCOT-HEART and CAPP trials) [24–26] and patients with negative troponin results and normal ECGs admitted for assessment of ACS (the CATCH and BEACON trials) [27–29] have been reported. All show promising results for the impact of CTCA on improving longer term clinical outcomes [30]. All consistently show a decreased rate of normal ICA, and the three studies reporting longer term outcomes are consistent in suggesting improved cardiovascular outcomes, including mortality, although these are all secondary analyses. None have been powered on relevant short or longer term important clinical outcomes.

However, early CTCA needs investigation in patients at intermediate risk for ACS, for whom improved and optimal targeting of interventions, including coronary revascularisation, and primary and secondary preventive therapy is likely to improve diagnosis and longer term outcome. The clinical and cost effectiveness of early CTCA in suspected or confirmed ACS must be clearly demonstrated before adoption of the technology into routine clinical practice, given its cost, risk and uncertainty of benefit. A positive or negative trial is equally important.

### The aims of the RAPID-CTCA trial

This study aims to investigate the effect of early CTCA in patients with suspected or confirmed ACS presenting to the ED, Medical Assessment Units (MAUs) or cardiology department, upon interventions, event rates and health care costs in a pragmatic clinical trial with economic evaluation up to 1 year after the trial intervention.

## Methods/design

**Fig. 1:**
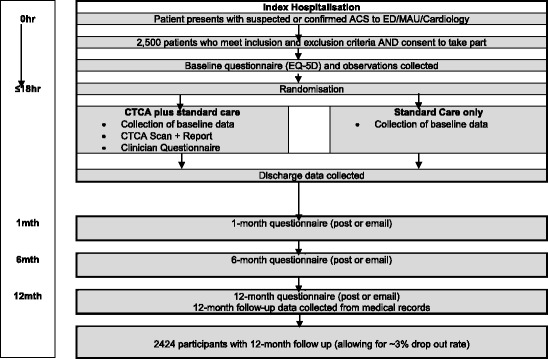
Patient pathway

RAPID-CTCA is an open, prospective, parallel-group, 1:1 randomised controlled trial of CTCA in addition to standard care (CTCA) versus standard care only (SCO) in adults presenting to the ED, MAU or cardiology department with suspected or confirmed ACS. Recruitment will take place in approximately 35 tertiary and district hospitals (with and without on-site ICA facilities) with ED, MAU, radiology and cardiology services. Participants allocated to the CTCA arm will receive the scan during the initial admission or, if discharged, as an ambulatory patient within 72 hours of randomisation. All participants will be followed up for 1 year. Figure 1 details the patient pathway from screening for eligibility to the end of follow-up.

### Primary endpoint

The primary endpoint will be all-cause death or recurrent non-fatal type 1 or type 4b MI at 1 year, measured as the time to the first such event. MI will be defined according to the most recent Universal Definition [13] and will be adjudicated by two independent cardiologists blinded to the intervention.

### Secondary endpoints

Table 1 describes the secondary endpoints.

**Table 1:** Secondary endpoints

1. Clinical, process and patient-centred endpoints	• Hospital length of stay • Coronary care length of stay • Proportion of patients receiving invasive coronary angiography (ICA) during index hospitalisation • Proportion of patients receiving coronary revascularisation during index hospitalisation • Proportion of patients receiving subsequent unplanned coronary revascularisation after index hospitalisation within 12 months • Proportion of patients in CTCA arm receiving ICA despite <50% stenosis on CTCA • Proportion of patients assigned to CTCA with normal or mild non-obstructive disease • Proportion of patients prescribed ACS therapies during index hospitalisation • Proportion of patients discharged on prevention treatment or who have alteration in dosage of prevention treatment during index hospitalisation • Representation or rehospitalisation with suspected ACS/recurrent chest pain within 12 months • Patient symptoms and quality of life up to 12 months • Health service resource utilisation • Patient satisfaction • Clinician certainty of presenting diagnosis after CTCA
2. Safety	• Proportion of patients with allergy/anaphylaxis/acute kidney injury • Proportion of patients with alternative diagnoses that relate to clinical presentation identified on CTCA, e.g. aortic dissection or pulmonary embolus • Proportion of patients with an incidental but potentially concerning finding on CTCA, e.g. malignancy or pulmonary nodules • Total average radiation exposure from CTCA in the intervention arm during index hospitalisation
3. Health economics	• Incremental cost per quality-adjusted life year (QALY) gained

### Population

The study population consists of patients ≥18 years old with symptoms mandating investigation for suspected or confirmed ACS with at least one of the following: ECG abnormalities, e.g. ST-segment depression >0.5 mm; history of ischaemic heart disease (where the clinician assessing patient confirms history based on patient history or available records); or troponin elevation above the 99^th^ centile of the normal reference range or increase in high-sensitivity troponin meeting ESC criteria for ‘rule-in’ of myocardial infarction (troponin assays will vary from site to site; local laboratory reference standards will be used). Table 2 lists the exclusion criteria.

**Table 2:** RAPID-CTCA trial exclusion criteria

1. Signs, symptoms or investigations supporting high-risk ACS	• ST elevation MI • ACS with signs or symptoms of acute heart failure • ACS with signs or symptoms of circulatory shock • Crescendo episodes of typical anginal pain • Marked or dynamic ECG changes, e.g. ST depression of >3 mm • Clinical team have scheduled early ICA on day of trial eligibility assessment
2. Patient inability to undergo CT	• Severe renal failure (serum creatinine >250 μmol/L or estimated glomerular filtration rate <30 mL/min/1.73 m^2^) • Contrast allergy • Beta blocker intolerance or allergy (if no alternative heart rate-limiting agent available/suitable) • Inability to hold breath • Atrial fibrillation (where mean heart rate is anticipated to be greater than 75 beats/min after beta blockade)
3. Patient had ICA or CTCA within the last 2 years revealing obstructive CAD or patient had ICA or CTCA within the last 5 years and the result was normal 4. Previous recruitment to the trial 5. Known pregnancy or currently breast feeding 6. Inability to consent 7. Further investigation for ACS would not be in the patient’s interest due to limited life expectancy, quality of life or functional status 8. Prisoners	

### Patient selection and enrolment

All potentially eligible patients are screened for eligibility by trained members of the research or clinical teams using triage information and clinical or electronic records in the ED, MAU or cardiology department. No additional trial-specific screening tests will be performed. The patient will receive routine acute clinical assessment including, as a minimum, a 12-lead ECG, vital signs measurement (pulse rate, non-invasive blood pressure, respiratory rate, conscious level, oxygen saturations and skin prick blood sugar) and admission routine blood tests including troponin and renal function. The results of these assessments will inform trial eligibility, and the patient may be approached as soon as they are available (normally in the first 2 hours after presentation). Patients may be recruited up to 18 hours after presentation. This time period has been chosen because it allows the longest period for recruitment where the patient could be deemed to be receiving acute assessment, i.e. up to the point where a 12-hour troponin result is being used by clinicians for acute decision making. Patient and clinician will be unaware of treatment allocation until after screening, consent and randomisation.

Eligible participants will be asked to provide written informed consent by appropriately trained and delegated members of the research or clinical team. After assessment for eligibility and consent, the clinical research nurse or a delegated member of the clinical team will collect the baseline data necessary to complete the pre-randomisation information. Table 3 provides a recommended schedule of enrolments, interventions and assessments for this trial.

**Table 3:** Template of recommended content for schedule of enrolment, interventions and assessments for RAPID-CTCA trial

		Study period					
	Enrolment	Allocation	Post-allocation				
Timepoint	*-t*_*1*_	0	≤72 h from randomisation	Discharge	1 month	6 months	12 months
Enrolment:							
Eligibility screen	X						
Informed consent	X						
Patient questionnaire	X						
Vital signs	X						
Data collection (secondary endpoints)	X	X					
Randomisation/allocation		X					
Interventions:							
CTCA (if randomised to CT arm)			X				
CTCA reporting (CT arm only)			X				
Clinician questionnaire (CT arm only)			X				
Assessments:							
Data collection (Primary/secondary endpoints)				X			X
Patient questionnaire (data collection secondary endpoints)					X	X	X

### Randomisation

Randomisation will be performed using a web-based randomisation service, managed by the Edinburgh Clinical Trials Unit (ECTU), that ensures allocation concealment. Once a patient is randomised, he/she will remain in the study and have all outcomes recorded regardless of compliance with randomised pathway allocation, unless the patient specifically withdraws consent to have data stored. Consented patients will be randomised on a 1:1 basis to CTCA in addition to standard care or standard care alone and will be stratified by study site. This is an open trial. The patient, recruiting and treating clinicians and radiologist will not be blinded to the intervention including the results of the CTCA. Outcome assessors, however, will be blinded to the intervention. Ineligible and non-recruited patients will receive standard medical care.

### Study interventions

CTCA will be delivered by a trained radiologist or cardiologist within an established radiology service. Patients randomised to standard care will receive the standard management for patients with suspected or confirmed ACS at that hospital site. The only difference will be the early use of CTCA in the intervention arm and the subsequent impact on patient care after the result is provided to the clinician for clinical decision making. Local chest pain management guidelines will be collected or confirmation obtained from the site principal investigator (PI) that CTCA is not currently in routine use for eligible patients (including change in practice during the period of the trial).

The technology being assessed is 64-slice, or greater, multidetector CT scanners enabled to perform ECG-gated cardiac studies. The examination may include a non-contrast ECG-triggered acquisition for calcium scoring (if part of the local protocol) and a post-contrast ECG-gated acquisition covering the whole of the heart and the root of the aorta.

The component of the total research protocol dose associated with the CTCA scan, i.e. that which is in addition to normal clinical care, is 10 mSv. This dose assessment is based on the dose to a typical patient. There will be normal variation around this average dose due to individual subjects’ body mass index (BMI) or heart rate or depending on whether or not a retrospective gating technique is used. In these circumstances, the dose may increase to approximately 30 mSv. Due to the variation in conversion factors used by sites to convert dose-length product (DLP) to effective dose in millisieverts, the radiation dose will be routinely reported for the trial as DLP. A DLP-to-millisieverts conversion factor of 0.014 mSv/mGy/cm will be used for trial reporting in line with recent publications in this area of research. A typical participant with a heart rate below 70 beats per minute in sinus rhythm and a BMI <25 should therefore have a DLP ≤714 mGy · cm; cases exceeding this value will be reported as a deviation. If at any participating centre the DLP regularly exceeds 1071 DLP mGy · cm, or any DLP exceeds 1428 mGy · cm, this will be reviewed to establish if a protocol deviation has occurred. All participating centres will be required to verify that their CTCA imaging protocol complies with the total research protocol dose prior to recruitment, and patient doses will be recorded and monitored as part of the study. An iodine-based contrast agent will be administered intravenously using the standard local procedure at each site. The use of glyceryl trinitrate (GTN) for coronary artery dilatation will be used at the discretion of individual centres.

CTCA will usually be reported by a trained radiologist or cardiologist at recruiting centres as soon as possible, ideally within 2 hours of the scan, and the results immediately communicated to the treating clinician. The clinical report detailing the results should be reported according to the Society of Cardiovascular CT guidelines, with the use of the American Heart Association coronary artery segment model, and will include both the calcium score if calculated and the presence of cardiac and non-cardiac findings. Stenoses will be quantified as no significant CAD (estimated stenosis <10%), mild non-obstructive CAD (estimated stenosis of 10–49%), moderate non-obstructive CAD (estimated stenosis of 50–70%) or obstructive CAD (estimated stenosis of >70%).

A proportion of CTCA reporting may be delivered remotely using remote access technology by a core group of readers. Transfer of image data is a well-established process within the UK for out-of-hours radiology reporting. Secure electronic transfer via the national Picture Archiving and Communication System (PACS) system (with required permissions) will allow reporting using voice recognition dictation either directly to the host radiology information system or via email directly to the referring centre.

A proportion of scans will be separately reported by experts independent to the trial and blinded to the initial report to measure inter-observer reliability. The first 10 scans performed at each site will undergo this process as well as a 5% random sample of the remaining scans.

### Impact of CTCA on participant management

CTCA results will be available to the clinical team to support acute clinical decision making. The provision of immediate CTCA with early reporting is expected to be variable across the centres. The intervention will be delivered by routine clinical staff. It is anticipated that 60–70% of patients will be recruited between 8.00 am and 6.00 pm and will be eligible for immediate CTCA [30]. Patients recruited to the study and randomised to CTCA will receive a CTCA as soon as feasible, and normally on the day of or day following randomisation, providing this does not significantly delay routine processes of care including discharge. Where a clinical decision is made to discharge the participant before the scan takes place, they will be asked to return for ambulatory CTCA within 72 hours and then be reviewed in the ED, MAU or cardiology department, depending on local procedures.

The results of the CTCA are likely to influence subsequent management. A guideline has been developed which will be available to clinicians to guide subsequent management dependent on the CTCA result (Appendix 1). A template letter is available to clinicians, if they wish to use it, to inform the patient’s primary care physician about non-obstructive CAD identified by the CTCA that may require secondary prevention being implemented by the primary care physician.

All other management and admission or discharge decisions will be at the discretion of the treating clinicians. Sites will be requested not to use CTCA as part of the routine investigation of ACS, and this will be closely monitored.

### Participant follow-up

Patients will normally be followed up using routine clinical notes and research contact directly with the patient by phone, email or post. At organisations where it is not possible to routinely get this data from administrative health service records, the research team at the site will attempt to contact the participant and/or their general practitioner (GP) by telephone to obtain the 12-month follow-up data. All patients will be followed up for 1 year.

Study participants are free to withdraw from the trial at any time. For patients who withdraw from active follow-up, routine data will continue to be collected unless the patient requests otherwise. The patient may be willing to give a reason for withdrawal, but this is not obligatory. Reasons, if given, will be recorded, and data collected up to that timepoint may be used in the final analyses, unless the patient specifically requests that their data not be used. If the patient withdraws consent to have their data stored, then this will be documented on the trial Consolidated Standards of Reporting Trials (CONSORT) flow diagram as ‘withdrawn’ and their data will not be used in the final analyses.

Any patient in the control group who has a CTCA as part of routine care within 30 days of randomisation will be defined as a crossover and will not be recorded as a deviation. Non-adherence will be defined as to have occurred in any participant not receiving a reported CTCA if randomised to it within 72 hours, and this would be recorded as a deviation. This allows ambulatory CTCA to be delivered when appropriate. Individual site retention, crossover and non-adherence will be monitored and reviewed at the Project Management Group (PMG) and Trial Steering Committee (TSC) meetings.

### Data collection

Data will be collected by the research team from routinely available administrative records or trial-specific documentation and will include the following categories: eligibility criteria, consent and baseline demographics, comorbidities, regular treatment, ECG results, vital signs, blood results, admission and discharge diagnoses, cardiology and other relevant investigations or interventions, length of stay, repeat hospitalisations and adverse events. Detail will also be collected on the trial intervention including timing, details of the procedure including dose, reporting clinician, report including incidental findings and any adverse events as a result of the intervention.

Length of stay and major adverse cardiac events will be recorded from telephone contact with patients, hospital and primary care records and death records from the Central Registry Office or equivalent. At baseline and at 1, 6 and 12 months, quality of life and angina symptoms will be measured using the EuroQol EQ-5D-5 L and Rose questionnaires by direct patient interview, postal or email survey with telephone follow-up for non-responders after two mailings 2 weeks apart.

### Statistical analysis and sample size calculation

One-year death or recurrent MI rate for this patient group is ~20% [11]. A total of 2424 evaluable patients are required (1212 per arm) to have 90% power to detect a 20% versus 15% difference in 1-year death or recurrent MI rate based on two-sided *p* < 0.05 using a chi-squared test. With a 3% drop-out rate, the sample size will be 2500 patients. Reviewing UK patient characteristics and presentation data, we estimate from previous data that there will be approximately 7000–8000 eligible patients annually across approximately 35 UK centres [11, 31, 32]. Although we anticipate and will aim for a higher recruitment rate than the 20% detailed below, our experience of emergency medicine trials has resulted in a conservative approach to estimation of recruitment rates. If we were to recruit 20% of these eligible patients and if all 35 centres recruited for 2 years, the trial would recruit about 3000 participants. We therefore believe, given the complexities of delivering this trial, that recruiting 2500 patients in about 35 centres over 2 years is feasible: about 1 patient per site, per week.

The trial will be reported on an intention-to-treat basis. The primary outcome is defined as first event of all-cause death or recurrent non-fatal MI type 1 or 4b. Time to primary outcome is defined as time from randomisation to primary outcome. Patients discontinuing the study (for any reason) prior to reaching primary outcome will have their time to primary outcome censored at the last contact date. The relationship between intervention and the primary outcome will be analysed using Cox proportional hazard regression adjusted for study site (used to stratify the randomisation), age, baseline Global Registry of Acute Coronary Events (GRACE) score, previous CAD and baseline troponin level. The results will be expressed as a hazard ratio with the corresponding 95% confidence intervals and *p* value. The individual elements of the composite primary outcome will be reported separately.

Subgroup analysis on the primary outcome is planned for age, baseline GRACE score, previous CAD and baseline troponin concentration. These will be assessed by examining the effect of entering the treatment by subgroup interaction into the Cox regression model. Secondary outcomes will be analysed using appropriate methods: logistic regression for binary outcomes and linear regression for normally distributed continuous outcomes, adjusted as described above. Continuous outcomes that are not normally distributed will be analysed using appropriate non-parametric techniques. The primary analysis will be intention-to-treat. Every effort will be made to minimise missing data, and our primary analysis will be a complete case analysis. If there is a sufficient level of missing data to affect our conclusions, a multiple imputation analysis will be undertaken, using clinically appropriate variables, as a sensitivity analysis. A full statistical analysis plan will be written during the trial and finalised prior to database lock.

### Economic analysis

Economic evaluation will assist policy makers in deciding whether multidetector CT scanning represents a cost-effective use of health service resources. The economic analysis will include (1) a within-trial cost effectiveness analysis (i.e. comparing the observed costs and QALYs of the intervention and control groups during the trial period) and (2) an analysis of the long-term cost effectiveness of CTCA, adapting an existing decision analytic model [18, 33].

In the within-trial cost-effectiveness analysis, incremental cost per QALY gained by using CTCA compared to standard care will be estimated by calculating the area under the curve for health utility using the EQ-5D-5 L and health service costs up to 1 year. Quality of life and angina symptoms will be measured using the EQ-5D-5 L and Rose questionnaires (see above) at baseline and at 1, 6 and 12 months after index admission. All health care consumption and costs will be estimated from a societal perspective using patient self-reported questionnaires and from hospital records. Costs will be attributed to the need for (1) continued hospitalisation, (2) additional invasive or non-invasive imaging, (3) drug therapy and (4) rehospitalisation for myocardial ischaemia. Costs of hospital admission will be measured using a top-down costing strategy. These costs will be measured for each patient in the trial and multiplied by national average costs to provide the estimated cost per patient. Local unit costs for staff and consumables will be obtained from each hospital finance department.

Long-term cost effectiveness will be estimated by adapting an existing model, developed as part of a previous evidence synthesis project [18, 32]. The model used published sources to capture patients’ life expectancy, annual costs and corresponding annual utilities, based on whether they had MI at initial hospital attendance and whether they suffered reinfarction. The data from the trial will be input into the model to estimate the lifetime QALYs and costs of surviving patients. The results will be reported as the incremental cost-effectiveness ratio (ICER) of the CTCA arm compared to usual care.

Sensitivity analyses will explore the potential impact of parameters upon costs, QALYs and ICERs. Parameter uncertainty will be included in a probabilistic sensitivity analysis based on Monte Carlo simulation. Cost-effectiveness acceptability curves (CEACs) will be plotted to identify the probability of the CTCA arm being cost effective compared to standard care for a range of willingness-to-pay values for an additional QALY.

### Trial oversight, ethics and governance

The trial has been reviewed and approved by the South East Scotland Research Ethics Committee (14/SS/1096). The Academic and Clinical Central Office for Research and Development (ACCORD) in Edinburgh is providing sponsorship and monitoring oversight for the project, and the trial will be conducted in line with the relevant sponsor standard operating procedures (SOPs). The ECTU is responsible for trial management, oversight of data collection and statistical analysis. The University of Sheffield is responsible for the health economics analysis. A PMG comprising the applicants and relevant members of the ECTU team will provide ongoing trial support. A writing committee will be formed by members of the PMG, and they will have responsibility for interpreting the data and writing and reviewing the final report. A draft of the final study report will be sent to the funder for peer review prior to submission for publication. A TSC and Data Monitoring Committee (DMC) have been established to oversee the safety, conduct and progress of the study. Appendix 2 details the RAPID-CTCA trial registration details. 

## Discussion

The RAPID-CTCA trial will recruit 2500 participants across about 35 hospital sites is the first study to investigate the role of CTCA in the early assessment of patients with suspected or confirmed ACS who are at intermediate risk, including patients who have raised troponin measurements during initial assessment. All previous ED trials have enrolled patients who are at low risk of ACS, supported by the exceptionally low subsequent 30-day and 1-year reported outcomes. Three recent trials in stable patients have demonstrated improved accuracy of diagnosis, an increase in downstream cardiological investigations including ICA and improved longer term cardiovascular outcomes in patients randomised to CTCA, although none of these trials was powered for these outcomes [18–20]. The CATCH trial in 600 patients with chest pain assessed for ACS in a single centre in Denmark further supports these findings with the recent reporting of the longer term clinical outcomes [26, 27]. They reported that CTCA was associated with better selection of patients for ICA with a lower rate of normal ICA results despite greater use of ICA. CTCA led to an increased use of preventative therapies and substantially more PCI at the index assessment. Lastly, a recently published trial from seven Dutch EDs did not show any short-term differences between standard care using high-sensitivity troponin assays alone or with the addition of CTCA, other than a reduction in downstream investigations [28]. This perhaps suggests that, with an optimised biomarker strategy in patients with no prior history of CAD or other higher risk features, CTCA is unlikely to affect clinical outcomes.

There have been a number of challenges in the set-up and delivery of the RAPID-CTCA trial. In the UK, the assessment and management of acute chest pain is truly multispecialty treatment (emergency medicine, acute and general medicine and cardiology), and this may vary considerably between sites and specialties within sites. In many centres, most patients receive no ancillary testing if they do not have high-risk features, and for many, ongoing care is provided by their primary care physician. Radiology access in terms of the types of investigations available, time of access and which specialties routinely have access to them also varies considerably between sites. This becomes even more complex in the context of a trial competing with other patients for scarce resources, in particular CT scanning within a target-driven system. Concerns regarding the risk of radiation exposure have also been an issue, especially as it has been perceived that, in this ’higher’ risk group, patients may receive radiation from both a CT and subsequent ICA. These issues have resulted in site engagement for the trial being far more complex and time-consuming than if the trial were being undertaken by a single specialty and has resulted in protracted set-up timelines and the placement of significant limits on recruitment due to resources.

If a trial of a diagnostic intervention such as CTCA is to result in change in clinical outcomes, it must influence downstream treatment. In patients with suspected or confirmed ACS this is likely to be due to higher rates of appropriate early coronary intervention after CT has defined the extent of disease, and in the longer term greater cardiological input and the use of preventative treatments for those patients with non-obstructive CAD.

Early CTCA needs investigation in intermediate-risk ACS patients where improved and optimal targeting of interventions, including coronary revascularisation, and primary and secondary preventive therapy is likely to improve diagnosis and longer term outcome. The clinical and cost effectiveness of early CTCA in suspected or confirmed ACS must be clearly demonstrated before adoption of the technology into routine practice, given its cost, risk and uncertainty of benefit. A positive or negative trial, therefore, is equally important.

### Trial status

The trial opened to recruitment in March 2015 with 34 UK sites participating in April 2016. Recruitment is anticipated to run until 30 June 2017 with trial completion by 31 December 2018. As of May 2016, 15% of the study population was recruited.

## Appendix 1

Table 4 presents the guideline for clinicians for subsequent management dependent on the CTCA result.

**Table 4:** Management guideline for trial intervention arm

CTCA result	Troponin result	Trial treatment recommendation
Obstructive disease: stenosis ≥70%	Positive or negative	1. ACS and secondary preventative therapies 2. Invasive coronary angiography (ICA) ± revascularisation
Moderate non-obstructive disease: stenosis 50–69%	Positive	1. ACS and secondary preventative therapies 2. Consider ICA if uncertainty about the presence of obstructive coronary artery disease or functional testing
Moderate non-obstructive disease: stenosis 50–69%	Negative	1. Secondary preventative therapies 2. Consider ICA if uncertainty about the presence of obstructive coronary artery disease or functional testing
Mild non-obstructive disease: stenosis <50%	Positive	1. Consider ACS and secondary preventative therapies 2. Consider alternative cause of chest pain and troponin rise
Mild non-obstructive disease: stenosis <50%	Negative	1. Discharge with no further follow-up 2. Consider secondary preventative therapies
Normal (no evidence of CAD)		Discharge with no further follow-up

## Appendix 2

Table 5 summarizes the registration details for this trial.

**Table 5:** Summary of key trial registration details

Data category	Information
Primary registry and trial identifying number	ISRCTN19102565
Date of registration in primary registry	03/10/2014
Secondary identifying numbers	NCT02284191
Source(s) of monetary or material support	National Institute for Health Research Health Technology Assessment Programme
Primary sponsor	Co-sponsored by University of Edinburgh and NHS Lothian (ACCORD)
Secondary sponsor(s)	N/A
Contact for public queries	rapid.ctca@ed.ac.uk
Contact for scientific queries	Alasdair.gray@nhslothian.scot.nhs.uk
Public title	Rapid Assessment of Potential Ischaemic Heart Disease with CTCA (the RAPID-CTCA trial)
Scientific title	The role of early CT coronary angiography in the evaluation, intervention and outcome of patients presenting to the emergency department with suspected or confirmed acute coronary syndrome
Countries of recruitment	UK
Health condition(s) or problem(s) studied	Acute coronary syndrome
Intervention(s)	CT coronary angiography
Key inclusion and exclusion criteria	INCLUSION CRITERIA Patient ≥18 years with symptoms mandating investigation for suspected or confirmed ACS with at least one of the following: ECG abnormalities, e.g. ST-segment depression >0.5 mm; history of ischaemic heart disease (where the clinician assessing patient confirms history based on patient history or available records); troponin elevation above the 99th centile of the normal reference range or increase in high-sensitivity troponin meeting European Society of Cardiology criteria for ‘rule-in’ of myocardial infarction (NB troponin assays will vary from site to site; local laboratory reference standards will be used). EXCLUSION CRITERIA 1. Signs, symptoms or investigations supporting high-risk ACS: ST elevation MI; ACS with signs or symptoms of acute heart failure or circulatory shock; crescendo episodes of typical anginal pain; marked or dynamic ECG changes, e.g. ST depression of >3 mm; clinical team have scheduled early invasive coronary angiography on day of trial eligibility assessment 2. Patient inability to undergo CT: severe renal failure (serum creatinine >250 μmol/L or estimated glomerular filtration rate <30 mL/min); contrast allergy; beta blocker intolerance (if no alternative heart rate-limiting agent available/suitable) or allergy; inability to hold breath; atrial fibrillation (where mean heart rate is anticipated to be greater than 75 beats/min after beta blockade) 3. Patient has had ICA or CTCA within last 2 years revealing obstructive coronary artery disease, or patient had either investigation within the last 5 years and the result was normal. 4. Previous recruitment to the trial 5. Known pregnancy or currently breast feeding 6. Inability to consent 7. Further investigation for ACS would not be in the patient’s interest, due to limited life expectancy, quality of life or functional status 8. Prisoners
Study type	Open parallel randomised controlled trial
Date of first enrolment	23/03/2015
Target sample size	2500
Recruitment status	Open
Primary outcome(s)	The primary endpoint will be all-cause death or recurrent non-fatal type 1 or type 4b MI at one year and time to first such event. MI will be defined according to the most recent Universal Definition [13] and will be adjudicated by two independent cardiologists blinded to the intervention
Key secondary outcomes	1. Hospital length of stay, coronary care length of stay 2. Proportion of patients receiving ICA during index hospitalisation 3. Proportion of patients receiving coronary revascularisation during index hospitalisation 4. Proportion of patients receiving subsequent unplanned coronary revascularisation after index hospitalisation within 12 months 5. Proportion of patients in CTCA arm receiving ICA despite <50% stenosis on CTCA 6. Proportion of patients assigned to CTCA with normal or mild non-obstructive disease 7. Proportion of patients prescribed ACS therapies during index hospitalisation 8. Proportion of patients discharged on prevention treatment or who have alteration in dosage of prevention treatment during index hospitalisation 9. Representation or rehospitalisation with suspected ACS/recurrent chest pain within 12 months 10. Patient symptoms and quality of life up to 12 months 11. National Health Service (NHS) resource utilisation 12. Patient satisfaction 13. Clinician certainty of presenting diagnosis after CTCA SAFETY 1. Proportion of patients with allergy/anaphylaxis/acute kidney injury 2. Proportion of patients with alternative diagnoses that relate to presentation on CTCA, e.g. aortic dissection or pulmonary embolus 3. Proportion of patients with incidental but potentially concerning finding on CTCA, e.g. malignancy or pulmonary nodules 4. Total average radiation exposure from CTCA in the intervention arm during index hospitalisation Cost effectiveness: estimated in terms of the incremental cost per quality-adjusted life year (QALY) gained

## Abbreviations

ACCORD: Academic and Clinical Central Office for Research and Development - Joint office for University of Edinburgh and NHS Lothian
ACS: Acute coronary syndrome
CABG: Coronary artery bypass graft
CAD: Coronary artery disease
CEACs: Cost-effectiveness acceptability curves
CT: Computerised tomography
CTCA: Computerised tomography coronary angiography
DMC: Data Monitoring Committee
ECG: Electrocardiogram
ECTU: Edinburgh Clinical Trials Unit
ED: Emergency department
EMERGE: Emergency Medicine Research Group, Edinburgh
ESC: European Society of Cardiology
GCP: Good Clinical Practice
GP: General practitioner
HTA: Health Technology Assessment
ICA: Invasive coronary angiography
ICER: Incremental cost-effectiveness ratio
IHD: Ischaemic heart disease
MAU: Medical Assessment Unit
MI: Myocardial infarction
PACS: Picture Archiving and Communication System
PCI: Percutaneous coronary intervention
PI: Principal investigator
PMG: Project Management Group
QALY: Quality-adjusted life year
ScHARR: School of Health and Related Research
SCO: Standard care only
SOP: Standard operating procedure
TSC: Trial Steering Committee
UK: United Kingdom
USA: United States of America
